# Host-associated *Bifidobacterium longum* exhibit gastrointestinal resilience and antagonistic activity against *Salmonella* serovars

**DOI:** 10.1016/j.crfs.2026.101580

**Published:** 2026-09-22

**Authors:** Bugil Choi, Yuting Zhai, Suyeun Noh, Jonathan Crabill, Katherine Kim, Miju Kim, Sangnam Oh, Younghoon Kim, Sam-Churl Kim, Min-Jin Kwak, Kwangcheol C. Jeong

**Affiliations:** a Emerging Pathogens Institute, University of Florida, Gainesville, FL, 32611, United States; b Department of Animal Sciences, IFAS, University of Florida, Gainesville, FL, 32611, United States; c Division of Applied Life Science (BK21Four, Institute of Agriculture & Life Science), Gyeongsang National University, Jinju, 52828, Republic of Korea; d Department of Food Science & Biotechnology, Kyung Hee University, Yongin, 17104, Republic of Korea; e Department of Functional Food and Biotechnology, Jeonju University, Jeonju, 55069, Republic of Korea; f Department of Agricultural Biotechnology and Research Institute of Agriculture and Life Science, Seoul National University, Seoul, 08826, Republic of Korea; g Department of Forest Products and Biotechnology, Kookmin University, Seoul, 02707, Republic of Korea

**Keywords:** *Bifidobacterium longum*, Host-associated probiotics, *Salmonella*, Antimicrobial activity

## Abstract

Probiotics are increasingly explored as alternatives to antibiotics for preventing and controlling infectious diseases in livestock, including enteritis, pneumonia, and sepsis. However, most commercially available probiotic strains originate from humans, which can limit their colonization efficiency and functional performance in livestock due to host-specificity. In this study, we isolated twelve *Bifidobacterium longum* strains from healthy dairy calves and evaluated their probiotic potential with a focus on gastrointestinal resilience and antimicrobial activity. Comparative genomic and phylogenetic analyses revealed that these bovine-derived isolates form a distinct genetic lineage, separated from previously reported human-, animal-, and food-derived strains, highlighting their host-specific evolution. *In vitro* stress tolerance assays showed that all isolates maintained high viability under simulated gastric fluid, low pH, bile salts, lysozyme exposure, and hyperosmotic conditions. The strains exhibited robust colonization and adhesion capability, based on cell surface hydrophobicity, auto-aggregation, mucin adhesion, and biofilm formation. Antimicrobial activity and competition assay indicated that several *B. longum* strains effectively inhibited *Salmonella enterica* serovars Typhimurium and Dublin. These findings indicate that host associated *B. longum* strains possess enhanced gastrointestinal survival and potent antagonistic activity against major enteric pathogens, thereby representing promising next-generation probiotic candidates for improving intestinal resilience.

## Highlights

• Host-associated probiotics may improve colonization and functional efficacy.
• Novel host-derived *B. longum* strains form a distinct genetic lineage.
• Strains show strong tolerance to simulated gastrointestinal stresses.
• Isolates display adhesion, aggregation, and biofilm formation traits.
• Selected strains inhibit *Salmonella* Typhimurium and Dublin.

## Graphical abstract

**Figure:**
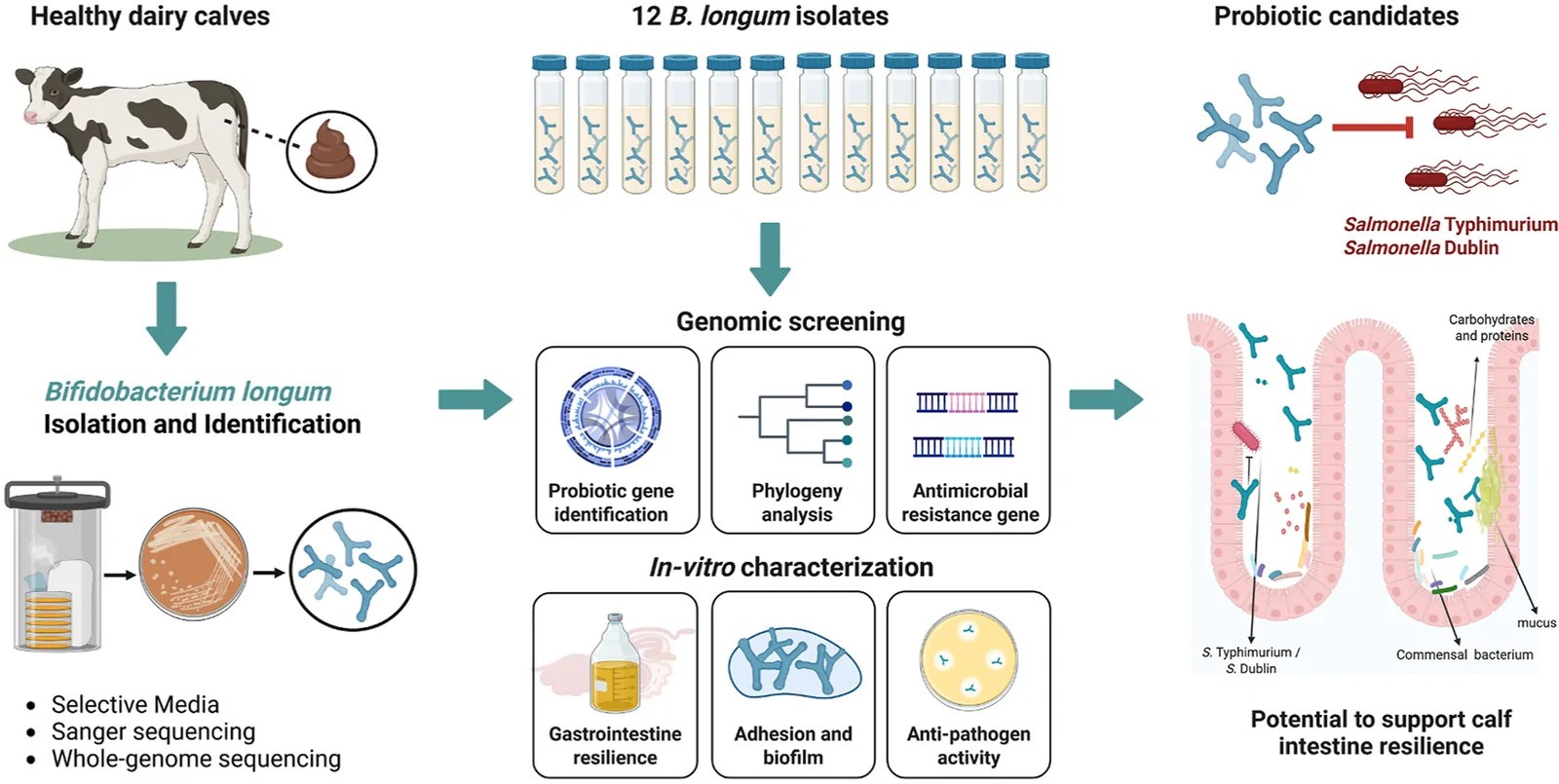

## 1 Introduction

Probiotics have gained increasing recognition as a sustainable strategy to enhance animal health and productivity in livestock systems, particularly as alternatives to antibiotic-based interventions (Kim et al., 2024; Zhai et al., 2024). By modulating gut microbiota composition, probiotics confer multiple benefits to host, including improved nutrient utilization, enhanced immune function, and optimized growth performance (Mafe et al., 2025; Srivastav et al., 2026). These effects are especially critical during early life stages, when the gastrointestinal tract (GIT) of young animals remain highly susceptible to pathogen colonization and dysbiosis, with long-lasting consequences for health and productivity (Xue et al., 2024). Probiotics supplementation during the weaning period has therefore been widely explored as a means to promote intestinal barrier maturation, regulate local and systemic immune responses, and reduce the incidence of gastrointestinal infections (Kwak et al., 2021).

Among the diverse probiotic candidates, *Bifidobacterium longum* is a well characterized Gram-positive, anaerobic bacterium, that has been extensively utilized as a human probiotic due to its capacity to modulate host immune responses, maintain intestinal barrier integrity, and inhibit the growth of enteric pathogens (Ku et al., 2024). The probiotic function of *B. longum* is largely mediated by the production of bioactive compounds, including short-chain fatty acids (e.g. acetate, propionate, and butyrate), extracellular polysaccharides, and bacteriocins, which contribute to anti-inflammatory effects and competitive exclusion of pathogens (Yu et al., 2025). In recognition of its favorable safety profile, *B. longum* has been granted Qualified Probable Safety status by the European Food Safety Authority and is classified as Generally Recognized as Safe by the U.S. Food and Drug Administration (Dong et al., 2024). As a result, *B. longum* has been widely incorporated into functional foods, dietary supplements, and therapeutic formulations targeting gastrointestinal disorders, primarily within the human health sector (Yoon et al., 2025). However, despite its proven efficacy in humans, the probiotic potential of *B. longum* strains derived from livestock remains comparatively underexplored.

Despite the widespread use of *B. longum,* the majority of currently available strains have been isolated from human sources, particularly infant feces, adult intestines, and breast milk (Yang et al., 2024). Consequently, human-derived probiotics may have limited efficacy in livestock, caused by reduced adhesion capacity and poor survival within the GIT, due to host-specificity driven by differences in gut anatomy, physiology, resident microbial community compositions, and diets (Zhang et al., 2025a). For example, oral administration of *B. longum* PCB133, originally isolated from infant feces, reduced *Campylobacter jejuni* counts in poultry but did not result in improved growth performance (Santini et al., 2010). Similarly, piglets administered a probiotic mixture containing *B. longum* subsp. *infantis* CECT 7210, an infant-derived strain, together with *Lactobacillus rhamnosus* HN001 failed to recover feed intake and weight following infection with enterotoxigenic *E. coli* (ETEC) F4 (Rodríguez-Sorrento et al., 2021). These findings suggest that the probiotic efficacy of *B. longum* strains may be strongly influenced by host origin and host-specific adaptation. Although recent studies using 16S rRNA gene sequencing and fecal microbiota transplantation have demonstrated that *Bifidobacterium*-dominated microbiota can promote host growth in murine models (Zhuang et al., 2024), the mechanistic understanding and functional characterization of *Bifidobacterium* isolates remain limited in livestock species. Therefore, we hypothesized that calf-derived *B. longum* strains from healthy dairy calves possess host-specific probiotic traits that enhance their survival and persistence in calves.

In this study, as an initial step toward developing calf-specific probiotics, we isolated and systemically characterized the probiotic potential of *B. longum* strains isolated from healthy calves. The isolates were evaluated using whole genome sequencing in combination with *in vitro* functional assays assessing gastrointestinal resilience, adhesion-related properties, and antimicrobial activity. These analyses were further complemented by *in silico* genomic approaches to identify genetic features associated with probiotic functionality.

## 2 Materials and methods

### 2.1 Animal selection and management

All animal procedures were reviewed and approved by the University of Florida Institutional Animal Care and Use Committee (Protocol #2019109709). Preweaning calves enrolled in this study were sourced from the University of Florida herd. The study included 47 healthy calves sampled between 3 and 28 days of age (Supplementary Table 1).

### 2.2 *Bifidobacterium* isolation and identification

A total of 47 fecal samples were collected from the rectal anal junction of healthy dairy calves. Approximately 2 g of feces per sample were obtained. Samples were resuspended in LB broth supplemented with 15% glycerol and stored at −80°C until processing. For *Bifidobacterium* isolation, fecal suspensions were plated on Bifidus Selective Medium (BSM) agar (Millipore Sigma, USA). Plates were incubated anaerobically at 37°C for 48 h using the GasPak EZ Anaerobe System (BD, USA). Up to six colonies were randomly selected from each BSM agar plate as a practical sampling strategy that balanced isolate representation with the feasibility of purification and downstream characterization. When fewer than six colonies were available, all colonies were selected. Selected colonies were purified on BSM agar for purification and preserved in 15% glycerol at −80°C. In total, 225 presumptive isolates were recovered from the 47 fecal samples. Of these, 72 isolates were selected for preliminary species identification by Sanger sequencing of the 16S rRNA gene. Selection was primarily stratified by sampling time point and calf identity; when possible, multiple isolates collected from the same calf on the same day were avoided to reduce repeated sampling of potentially closely related isolates. Based on the 16S rRNA gene identification results, 16 *Bifidobacterium longum* isolates were selected for whole-genome sequencing to represent different calves and sampling ages. Whole-genome-based taxonomic classification confirmed 12 isolates as *B. longum*, which were included in the subsequent genomic and *in vitro* probiotic characterization. Genomic DNA was extracted from purified isolates using the DNeasy Blood and Tissue Kit (Qiagen, USA) following the manufacturer's protocol for Gram-positive bacteria. Species identification was performed by amplifying the 16S rRNA gene using universal primers KCP812 (5′-CAG GCC TAA CAC ATG CAA GTC-3′) and KCP813 (5′-GGG CGG WGT GTA CAA GGC-3′). Purified PCR products were sequenced by Genewiz (South Plainfield, NJ, USA). Resulting sequences were compared against the NCBI 16S rRNA database using BLAST. Whole-genome libraries were prepared using the Nextera XT DNA Library Prep Kit (Illumina, USA). Sequencing was performed on an Illumina NextSeq 1000 platform using a P1 cartridge (2 × 300 bp). Raw reads were quality-trimmed using Sickle, applying a quality threshold of Q30 and minimum read length of 50 bp. Trimmed reads were assembled with SPAdes (Bankevich et al., 2012) using k-mers of 21, 33, 55, 77, 99, and 127. Mean sequencing depth was determined by mapping the quality-filtered paired-end reads to their corresponding assemblies using BWA-MEM v0.7.17 (Li and Durbin, 2009), followed by alignment processing and depth calculation using SAMtools v1.20 (Li et al., 2009). Assembly statistics, including total assembly length, contig number, largest contig, N50, L50, and GC content, were calculated using QUAST v5.3.0 (Mikheenko et al., 2018). Genome completeness and contamination were estimated using CheckM2 v1.1.0 (Chklovski et al., 2023). Complete strain-level quality metrics are provided in Supplementary Table 1. PubMLST-based whole-genome classification was used for definitive species identification.

### 2.3 Bacterial genome annotation and phylogeny

Whole-genome annotation of the 12 *B. longum* isolates was performed using Prokka v1.14.6 (Seemann, 2014). Antimicrobial resistance genes (ARGs) and virulence factors (VFs) were identified using Abricate v1.0.1 (Seemann, 2016) with the CARD (Alcock et al., 2023) and VFDB (Chen et al., 2005) databases, respectively. To evaluate bacteriocin biosynthetic potential, assembled genomes were screened using BAGEL4 (van Heel et al., 2018), with default parameters. No known bacteriocin biosynthetic gene clusters were detected. Putative probiotic-associated genes were selected based on previously published literature and identified across genomes using Roary (Alessandri et al., 2021; van Kranenburg et al., 1999; Ruiz et al., 2017a; Page et al., 2015). Protein similarity was compared using ClustalW alignment in MEGA v11 for Microsoft Windows, and maximum-likelihood phylogenies of protein sequences were constructed accordingly (Tamura et al., 2021). Pan-genome comparison and visualization of the *B. longum* strains were applied using Proksee (Grant et al., 2023).

Core-genome alignment of the *B. longum* isolates was generated using Roary v3.13.0 and phylogenetic reconstruction was performed with IQ-TREE v3.0.1 using ModelFinder to determine the best-fit substitution model and 1000 bootstrap replicates (Minh et al., 2020). To compare these isolates with publicly available *B. longum* strains, we downloaded genomes from the NCBI RefSeq database. A core-genome phylogeny including both our isolates and reference strains was generated using the same workflow described above. Both phylogenetic trees were visualized using iTOL v7.4.2 (Letunic and Bork, 2024).

### 2.4 Bacterial culture

*B. longum* strains were recovered from frozen stocks and streaked onto De Man, Rogosa and Sharpe agar (MRS; BD Difco™, Sparks, MD, USA) supplemented with 0.05% L-cysteine (MRSc; Thermo Scientific, Waltham, MA, USA) and incubated anaerobically at 37°C for 48 h. A single colony was then inoculated into MRSc broth and incubated anaerobically for 48 h at 37°C using AnaeroPack™ (MGC, Tokyo, Japan), with the addition of 1 mL sterile mineral oil (Thermo Scientific, Waltham, MA, USA). A 1:10 dilution was subsequently prepared to establish main cultures for downstream experiments. *Salmonella enterica* serovar Typhimurium KCJ187 and *S. enterica* serovar Dublin KCJ2K3839 were streaked onto Tryptic Soy Agar (TSA; BD Difco™, Sparks, MD, USA) plates and incubated at 37°C for 18 h. A single colony was then inoculated into 5 mL Tryptic Soy Broth (TSB; BD Difco™, Sparks, MD, USA) to prepare seed cultures. Main cultures were subsequently obtained by transferring a 1:100 dilution into fresh 5 mL TSB and incubated aerobically overnight at 37°C with shaking at 200 rpm.

### 2.5 Simulated gastric fluid tolerance

Simulated gastric fluid (SGF) tolerance of *B. longum* strains was determined according to the method described by Polzin et al. (2013). Briefly, SGF solution was prepared with 0.5% (w/v) pepsin from porcine gastric mucosa (Sigma-Aldrich, St. Louis, MO, USA), 0.5% (w/v) NaCl (Thermo Scientific Waltham, MA, USA), and adjusted pH to 3 using 6 N HCl. The solution was sterilized using a 0.22 μm filter. Forty-eight-hour *B. longum* cultures were adjusted to an OD_600_ of 1. The initial viable cell count (0 h) was determined by serial dilution and plating on MRSc agar. For the SGF tolerance assay, 100 μL of the same culture was inoculated into 900 μL of SGF and incubated at 37°C for 2 h under anaerobic conditions with 1 mL mineral oil. Following incubation, the bacterial suspension was serially diluted, plated on MRSc agar, and incubated anaerobically at 37°C for 48 h. Viable colonies were enumerated and the survival rate was expressed as a percentage by dividing the viable cell count after 2 h of SGF exposure by the initial viable cell count before exposure and multiplying by 100.

### 2.6 Acid tolerance

The acid tolerance of *B. longum* strains was determined at low pH conditions (Massip et al., 2019). Forty-eight-hour *B. longum* cultures were adjusted to an OD_600_ of 1. The initial viable cell count (0 h) was determined by serial dilution and plating on MRSc agar. For the acid tolerance assay, 100 μL of the same culture was inoculated into 900 μL of MRSc adjusted to pH 3 and incubated for 1 or 2 h under anaerobic conditions with a mineral oil overlay. After 1 and 2 h of acid exposure, the bacterial cultures were serially diluted, plated on MRSc agar, and incubated anaerobically at 37°C for 48 h. Viable colonies were enumerated and the survival rate at each time point was expressed as a percentage by dividing the viable colony count after 1 or 2 h of acid exposure by the initial viable colony count at 0 h and multiplying by 100.

### 2.7 Bile salt tolerance

Bile salt tolerance of *B. longum* strains was evaluated as previously described (Zhai et al., 2024), using in MRSc supplemented with 0.2% bile salt (Sigma-Aldrich, St. Louis, MO, USA). Forty-eight-hour *B. longum* cultures were adjusted to an OD_600_ of 1. The initial viable cell count (0 h) was determined by serial dilution and plating on MRSc agar. For the bile salt tolerance assay, 100 μL of the same culture was inoculated into 900 μL of bile salt medium and incubated for 4 h under anaerobic conditions with a mineral oil overlay. After 4 h of bile salt exposure, the bacterial cultures were serially diluted, plated on MRSc agar, and incubated anaerobically at 37°C for 48 h. Viable colonies were enumerated and the survival rate was expressed as a percentage by dividing the viable colony count after 4 h of bile salt exposure by the initial viable colony count at 0 h and multiplying by 100.

### 2.8 Lysozyme tolerance

Lysozyme tolerance of *B. longum* strains was determined as previously described (Rabaoui et al., 2023). Forty-eight-hour *B. longum* cultures were adjusted to an OD_600_ of 1 and washed twice with 1X phosphate-buffered saline (PBS). The washed bacterial cells were centrifuged (2000 × *g*, 10 min) and resuspended in 2 mL of ringer solution ((CaCl_2_ 0.03 g/L (Thermo Scientific, Waltham, MA, USA), NaCl 1.5 g/L (Thermo Scientific, Waltham, MA, USA), KCl 0.02 g/L (Sigma-Aldrich, St. Louis, MO, USA), and NaHCO_3_ 0.03 g/L (Thermo Scientific, Waltham, MA, USA)). Subsequently, 100 μL of the bacterial suspension was inoculated into 900 μL of electrolyte solution (CaCl_2_ 0.22 g/L, NaCl 6.2 g/L, KCl 2.2 g/L, and NaHCO_3_ 1.2 g/L) with (ESL) or without (ES) lysozyme (100 mg/L, Thermo Scientific, Waltham, MA, USA) with mineral oil overlay. ES and ESL cultures were incubated anaerobically at 37°C for 2 h. Following incubation, the bacterial cultures were serially diluted, plated on MRSc agar, and incubated anaerobically at 37°C for 48 h. Viable colonies were enumerated and the survival rate was expressed as a percentage by dividing the viable colony count after 2 h of lysozyme exposure by the initial viable colony count at 0 h and multiplying by 100.

### 2.9 Osmolarity tolerance

Osmolarity tolerance of *B. longum* strains was determined as previously described (Fugaban et al., 2021). Forty-eight-hour *B. longum* cultures were adjusted to an OD_600_ of 1. One milliliter of 1 OD_600_ *B. longum* cultures was washed twice and resuspended in MRSc medium. Subsequently, 100 μL of the bacterial suspension was inoculated into MRSc medium supplemented with 1%, 2%, or 3% NaCl and incubated anaerobically at 37°C for 48 h with mineral oil overlay. Bacterial growth was measured at OD_600_ using a spectrophotometer.

### 2.10 Cell surface hydrophobicity

Cell surface hydrophobicity of *B. longum* strains was determined as previously described (Lei et al., 2024; Cheruvari and Kammara, 2024). Forty-eight-hour *B. longum* cultures were harvested, washed twice, and resuspended in 1X PBS to an OD_600nm_ of 1. A 3 mL of bacterial suspension was mixed with 1 mL of xylene (Sigma-Aldrich, St. Louis, MO, USA) and vortexed for 2 min to facilitate interaction between bacterial cells and the hydrocarbon phase. The mixture was then incubated at room temperature for 1 h to allow phase separation. The OD600 of the aqueous phase was measured before (OD_initial) and after (OD_final) xylene treatment. Cell surface hydrophobicity was expressed as a percentage and calculated as [(OD_initial – OD_final)/OD_initial] x 100.

### 2.11 Auto-aggregation

Auto-aggregation ability of *B. longum* strains was determined according to a previously described method (Angulo et al., 2025). Briefly, bacterial cultures were harvested, washed twice with 1X PBS, and resuspended in PBS to an OD_600nm_ of 1.0. The bacterial suspension was mixed thoroughly, and the initial absorbance was measured at 600 nm (OD_initial). The suspension was then incubated at room temperature without disturbance for 5 h. At 1 h intervals, 1 mL of the upper phase of the suspension was carefully collected, and the OD_600_ (OD_t) was measured to calculate auto-aggregation percentage. Auto-aggregation was expressed as a percentage and calculated as [(OD_initial – OD_t)/OD_initial] x 100.

### 2.12 Mucin adhesion and biofilm formation

Mucin adhesion of *B. longum* strains was evaluated according to a previously described method (Leccese Terraf et al., 2014). Briefly, 100 μL of porcine stomach mucin (Type III, Sigma-Aldrich, St. Louis, MO, USA) at 1 mg/mL in 1X PBS was added to each well of a polystyrene 96-well microplate and incubated overnight at 4°C to allow coating. The wells were then washed twice with 1X PBS to remove unbound mucin. *B. longum* culture was harvested, washed, and resuspended in PBS to an OD_600_ of 1.0. Subsequently, 100 μL of the resuspended bacterial culture was added into the mucin-coated well and incubated at 37°C for 2 h without shaking under the anaerobic condition by mineral oil overlay. After incubation, wells were washed three times with 100 μL of 1X PBS to remove unattached bacteria. The attached cells were stained with 100 μL of 0.1% crystal violet for 15 min, followed by washing twice with 1X PBS to remove excess stain. The bound crystal violet was eluted with 100 μL of 95% ethanol and the absorbance was measured at 595 nm using a microplate reader to evaluate the mucin adhesion ability of *B. longum* strains.

Biofilm formation may contribute to the persistence and colonization of probiotic bacteria in the GIT. Biofilm formation of *B. longum* strains was evaluated according to a previously described method (Espeche et al., 2009). Briefly, 200 μL of mucin was coated onto each well of a 96-well plate and washed as described in the mucin adhesion assay. *B. longum* cultures were washed twice with 1X PBS and resuspended in MRSc media to an OD_600_ of 0.4. Subsequently, 200 μL of the bacterial suspension was added to each mucin-coated well and incubated anaerobically at 37°C for 48 h without shaking. Anaerobic conditions were maintained using a mineral oil overlay. Following incubation, wells were washed three times with 200 μL of 1X PBS to remove unattached bacteria. The attached biofilm was stained with 100 μL of 0.1% crystal violet for 15 min, washed with 1X PBS, and the bound dye was eluted with 100 μL of 95% ethanol. The absorbance was measured at 595 nm using a microplate reader to quantify biofilm formation.

### 2.13 Antimicrobial activity assay

Antimicrobial activity of *B. longum* strains was evaluated using the agar well diffusion method as previously described (Boranbayeva et al., 2020). Soft tryptic soy agar (sTSA) was prepared using 30.0 g/L of TSB and 7.5 g/L of agar. *S.* Typhimurium KCJ187 or *S.* Dublin KCJ2K3839 strain was inoculated into sTSA to a final concentration of 10^7^ CFU/mL, and 15 mL of sTSA was poured into sterilized Petri dishes and allowed to solidify. After solidification, 6-mm wells were created in the agar. The overnight culture of *B. longum* or *Salmonella* strains was centrifuged (2000 × *g*, 10 min), and the cell-free supernatant was collected. To obtain neutralized cell-free supernatant, the pH of the supernatant was adjusted to 7.0 with NaOH, followed by a filtration through a 0.22 μm filter. Subsequently, 50 μL of the supernatant was added to each well and allowed to diffuse for 2 h at room temperature in a fume hood. An additional 50 μL of the supernatant was added to the same wells. The plates were incubated overnight at 37°C under aerobic conditions. After incubation, the presence of a clear inhibition zone surrounding the well was considered indicative of antimicrobial activity of *B. longum* strains. Supernatant from *Salmonella* cultures was used as the negative control.

### 2.14 Bacterial competition assay

Bacterial competition assays were performed according to a previously described method (Zhai et al., 2024). Stationary phase cultures of *B. longum* strains and *S.* Typhimurium KCJ187 and *S.* Dublin KCJ2K3839 were used to evaluate the inhibitory effect of *B. longum* strains during co-culture. Both probiotic and pathogen cultures were adjusted to OD_600_ of 1, washed with 1X PBS, and resuspended in MRSc media. Subsequently, 10 μL of each culture was incubated into 2 mL of fresh MRSc medium and co-incubated at 37°C for 48 h under anaerobic conditions maintained by overlaying with 1 mL of mineral oil. After incubation, the cultures were serially diluted and plated onto TSA plates, followed by overnight incubation at 37°C to enumerate the number of surviving *Salmonella* cells affected by co-incubation. For the negative control, 10 μL of pathogen culture resuspended in MRSc was incubated under the same conditions without *B. longum.*

### 2.15 Statistical analysis

Statistical analyses were performed using GraphPad Software Prism version 10.1.1. Differences among *B. longum* strains were analyzed using one-way analysis of variance (ANOVA) followed by Tukey's multiple comparison test for tolerance to SGF, low pH, bile salts, lysozyme, cell surface hydrophobicity, mucin adhesion, and biofilm formation. The antimicrobial activity and bacterial competition assay against *Salmonella* strains was analyzed using one-way ANOVA followed by Dunnett's multiple comparison test, comparing the number of surviving *Salmonella* cells with the negative control. A *P*-value < 0.05 was considered statistically significant.

## 3 Result

### 3.1 *Bifidobacterium* isolation and probiotic potential characterization

As *Bifidobacterium* has been widely recognized for its probiotic potential and its ability to beneficially modulate the gut microbiome in animals, in this study, we aimed to isolate and characterize *Bifidobacterium* strains from healthy dairy calves. Rectal fecal samples were collected from calves between Day 3 and Day 28 after birth, yielding a total of 16 isolates recovered using selective media and 16S rRNA gene sequencing. Whole-genome sequencing and taxonomic classification identified 12 isolates as *Bifidobacterium longum*, three as *Bifidobacterium pseudocatenulatum*, and one isolate as *Ligilactobacillus salivarius*. Core-genome phylogenetic analysis revealed that the *B. longum* isolates were genetically close overall but formed two distinct clades containing five and seven isolates, respectively (Fig. 1A). No identical genomes were detected, indicating strain-level diversity among isolates. Strains in clade 1 highlighted in orange were closely similar, except for isolate KCJ2K3574, which originated from a 3-day-old calf and showed relatively greater genetic divergence. Notably, all strains isolated from Day 9 calves grouped within clade 2 highlighted in blue, suggesting possible age-related colonization of *B. longum* in early-life calves.

**Fig. 1:**
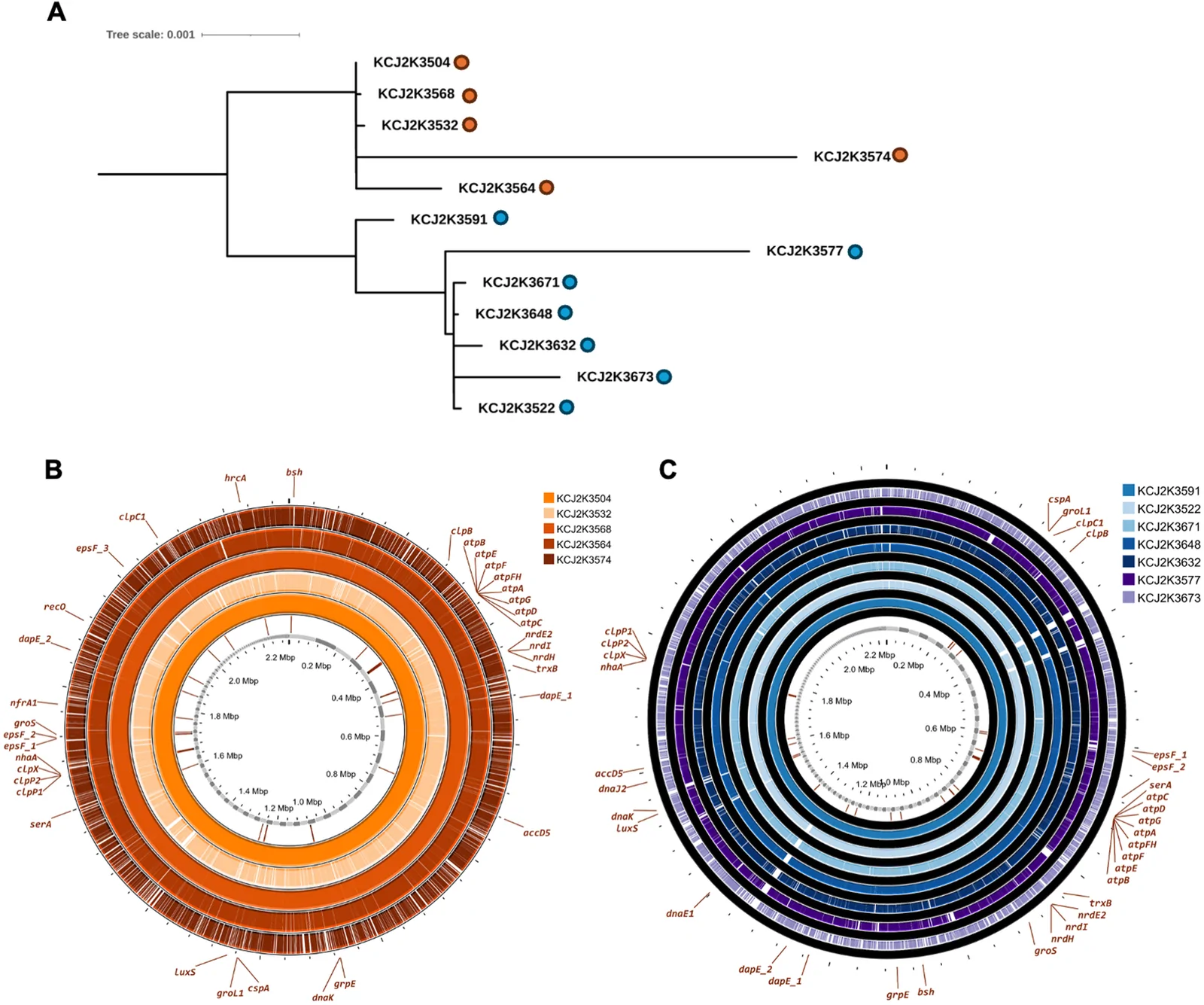
**Phylogenetic relationships and distribution of probiotic-associated genes among *Bifidobacterium longum* isolates from dairy calves.** (A) Core-genome phylogeny of 12 *B. longum* isolates isolated from healthy dairy calves, showing two phylogenetic clusters highlighted in orange and blue. (B–C) Genome maps of strains from each phylogenetic cluster illustrating the distribution of annotated probiotic-associated genes, including genes associated with acid tolerance, exopolysaccharide biosynthesis, biofilm formation, and stress response. Strains KCJ2K3504 and KCJ2K3591 were used as reference genomes for the orange (B) and blue clusters (C), respectively.

It is known that genes involved in acid tolerance, biofilm formation, adhesion capacity, and stress resilience are associated with probiotic functions of *B. longum* (Alessandri et al., 2021; van Kranenburg et al., 1999; Ruiz et al., 2017b). To identify genes related to probiotic traits, we conducted genome annotation. As shown in Fig. 1B for cluster 1 and Fig. 1C for cluster 2, all strains encode multiple genetic determinants associated with probiotic functionality. The absence and presence of each gene related to probiotic functions are summarized in Table 1. Genes associated with acid tolerance mediated by the ATP synthase complex encoded by the *atpA-H* gene cluster contribute to intracellular pH homeostasis under acidic conditions and support bacterial survival in the GIT. Genes related to biofilm formation were also widely distributed, with approximately twenty loci involved in exopolysaccharide (EPS) biosynthesis and biofilm assembly identified across strains. Among these, multiple *epsF* variants were observed, displaying strain-specific sequence variation. Glycosyltransferases encoded within EPS gene clusters are key enzymes responsible for assembling repeating polysaccharide units, which may influence bacterial adhesion, surface colonization, and persistence within the host gut. Components of the ribonucleotide reductase system, including *nrdH*, *nrdI*, and *nrdE2*, were also conserved among strains. These genes support deoxyribonucleotide synthesis and contribute to genome stability, particularly under environmental and oxidative stress conditions. Several ATP-dependent chaperone–protease systems, including *clpX*, *clpB*, *clpP*, and *clpC*, were detected across all genomes, indicating conserved mechanisms for protein quality control and cellular stress adaptation. In addition, heat shock proteins encoded by *dnaK* and *dnaJ*, together with the *groL*/*groS* chaperonin system, were present in most of the strains (9/12), further supporting the capacity of these strains to tolerate environmental and GIT stresses. Together, these genomic characteristics suggest that the isolates possess probiotic potential.

**Table 1:** Presence and absence of probiotic associated genes.

	Genes	KCJ2K3504	KCJ2K3522	KCJ2K3532	KCJ2K3564	KCJ2K3568	KCJ2K3574	KCJ2K3577	KCJ2K3591	KCJ2K3632	KCJ2K3648	KCJ2K3671	KCJ2K3673
**Acid resistance**	***atpA***	1	1	1	1	1	1	1	1	1	1	1	1
	***atpB***	1	1	1	1	1	1	1	1	1	1	1	
	***atpC***	1	1	1	1	1		1	1	1	1	1	
	***atpD***	1	1	1	1	1		1	1	1	1	1	
	***atpE***	1	1		1	1	1	1	1		1	1	1
	***atpF***	1	1		1	1		1	1		1		
	***atpFH***	1	1	1	1	1		1	1	1	1	1	
	***atpG***	1	1	1	1	1		1	1		1	1	
**Biofilm**	***nhaA***	1		1	1	1	1	1	1	1			1
	***accD5***	1	1		1	1	1	1	1	1	1	1	
	***dapE_1***	1	1		1	1	1	1	1		1	1	
	***dapE_2***	1	1		1	1	1	1	1	1	1	1	1
	***epsF***		1						1	1	1	1	
	***epsF_2***	1			1	1							
	***epsH***							1					
	***epsJ_1***							1					
	***epsJ_2***							1					
	***epsJ_3***							1					
	***epsF_3(group_1776)***	1			1	1							
	***epsF_2 (group_2642)***	1			1	1							
	***epsF_2 (group_2787)***		1						1		1		
	***epsF_1 (group_3938)***							1					
	***epsF_2 (group_4056)***							1					
	***luxS***	1	1		1	1	1		1		1		
	***nrdE2***	1	1	1	1	1	1	1	1	1	1	1	1
	***nrdH***	1	1	1		1		1	1	1	1	1	1
	***nrdI***	1	1	1	1	1		1	1	1	1		1
	***serA***	1	1			1		1	1	1	1	1	1
**Stress tolerance**	***bsh***	1	1	1	1	1	1	1	1	1	1	1	1
	***clpB***	1	1		1	1		1	1	1	1	1	1
	***clpC1***	1		1	1	1		1	1		1		
	***clpP***			1									
	***clpP1***	1	1		1	1	1	1	1	1	1		1
	***clpP2***	1	1			1	1	1	1	1	1	1	
	***clpX***	1	1	1	1	1	1	1	1	1	1	1	
	***cspA***	1	1		1	1	1	1	1		1	1	1
	***dnaJ2***	1	1		1	1	1	1	1		1		
	***dnaK***	1	1	1	1	1	1	1	1	1	1	1	
	***dnaK_2***							1					
	***groL1***	1			1	1	1	1	1	1	1	1	
	***groS***	1	1	1	1	1		1	1		1	1	
	***grpE***	1	1	1		1		1	1	1	1	1	
	***trxB***	1	1	1	1	1	1	1	1	1	1	1	

### 3.2 Phylogenetic distinction of *B. longum* strains isolated from healthy calves

To assess the phylogenetic relationships between calf-derived isolates and strains from other sources, a core-genome phylogeny was constructed using 792 publicly available *B. longum* genomes obtained from diverse hosts. Most publicly available genomes originated from humans (n = 759), followed by food-associated sources (n = 14), cows (n = 9), pigs (n = 5), dogs (n = 4), and cats (n = 1). Host-associated clustering patterns were observed within the phylogenetic tree (Fig. 2A). Interestingly, strains sourced from livestock, including dairy calves in this study, as well as cows and pigs, tended to cluster together (marked with asterisk), whereas strains isolated from companion animals and food sources were more interspersed among human-derived strains. These data indicate potential host-associated selection of *B. longum*. Notably, the 12 calf-derived *B. longum* isolates formed a distinct cluster within the livestock-associated clade (Fig. 2B). This data supports host-associated diversification and suggests that colonization and persistence of *B. longum* strains may be influenced by host-specific ecology and gut environments. Overall, these results highlight host-associated genomic variation within *B. longum* and emphasize the importance of considering host specificity when developing probiotics for livestock applications.

**Fig. 2:**
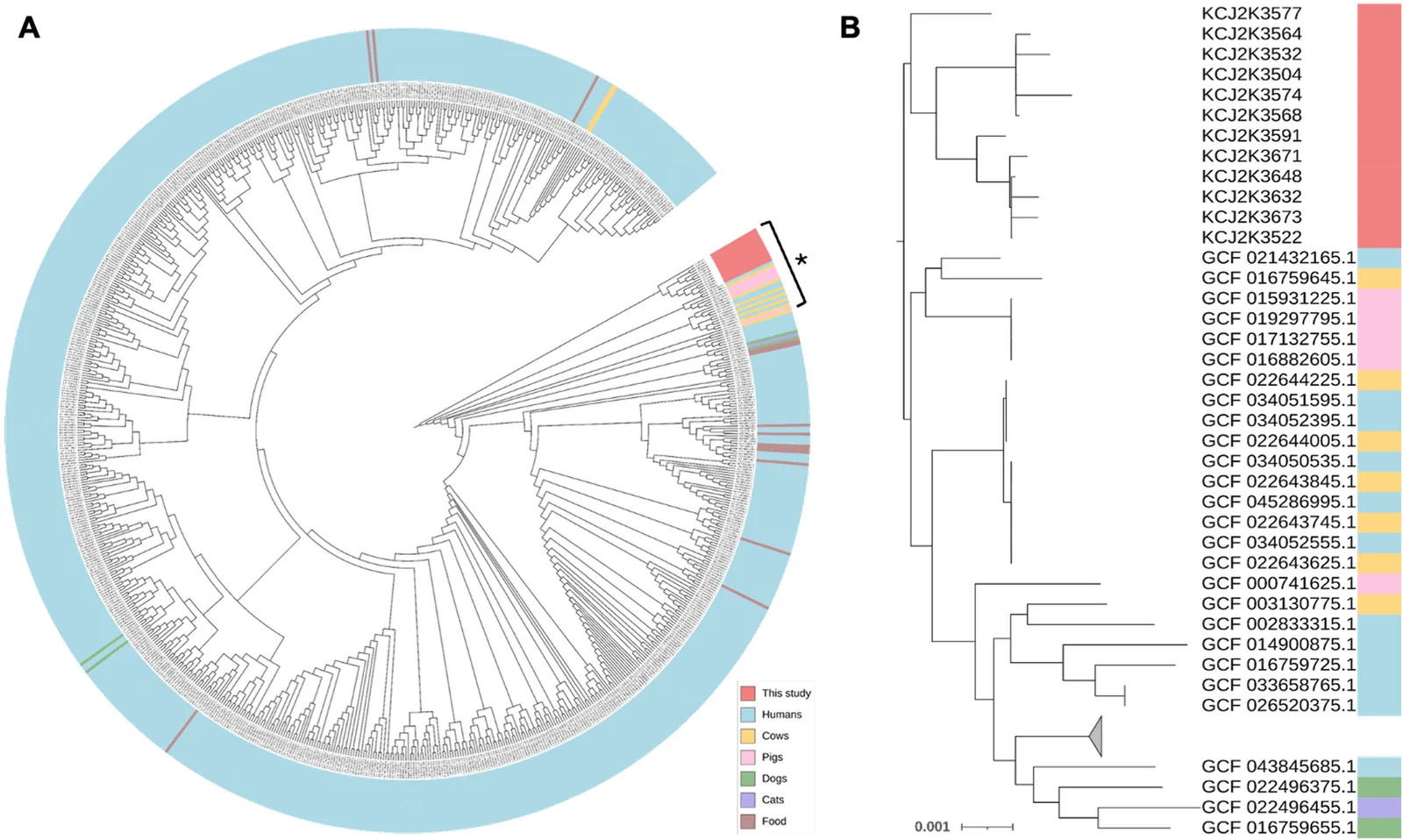
**Phylogenetic relationships of *Bifidobacterium longum* isolates from this study among publicly available genomes.** (A) Maximum-likelihood phylogenetic tree constructed from a core-genome alignment of 804 *B. longum* genomes, including 792 publicly available genomes and 12 isolates from this study isolated from healthy calves. The outer colored ring indicates the host origin of each isolate. The isolates obtained in this study are highlighted in red, whereas publicly available genomes are colored according to their source, including humans, cows, pigs, dogs, cats, and food. The bracketed region marked with an asterisk (*) denotes the clade containing the isolates from this study. (B) Expanded view of the clade highlighted in (A), showing detailed phylogenetic relationships between isolates from this study and closely related reference genomes. The gray triangular clade represents 765 genomes predominantly originating from humans. Tip labels correspond to genome accession numbers, and the scale bar indicates substitutions per site. The 12 *B. longum* isolates from this study form distinct clusters relative to other genomes.

### 3.3 Viability of *B. longum* strains isolated from healthy calves under gastrointestinal environments

Because probiotic functionality is critically dependent on survival during GIT passage, the viability of *B. longum* isolates was assessed under simulated gastrointestinal stress conditions. The isolates were exposed to various stressors relevant to the GIT, including pepsin-containing gastric fluid, acidic pH, bile salts, lysozyme, and osmotic pressure.

To assess resistance to gastric conditions, bacterial viability was first evaluated in simulated gastric fluid (SGF), containing 0.5% pepsin with 0.5% NaCl, designed to mimic the enzymatic and acidic environment of the stomach (Fig. 3A). After 2 h of incubation in SGF, all tested strains, excepting KCJ2K3632 and KCJ2K3648, exhibited great viability (>10% viability). Nonetheless, all strains showed viability above 10^7^ CFU/mL, indicating their potential to survive during GIT passage and reach the small intestine.

**Fig. 3:**
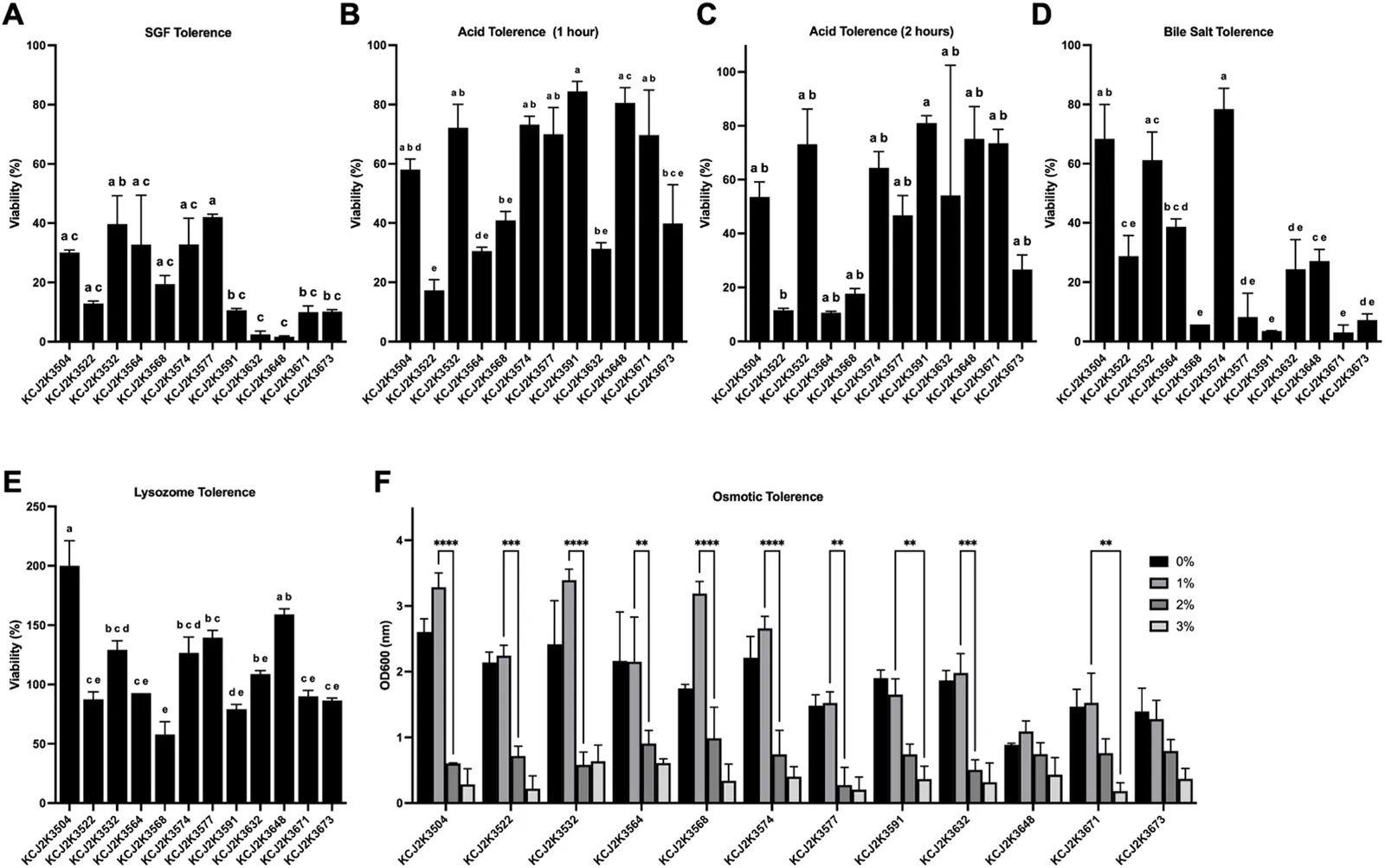
**Viability of *B. longum* strains under simulated gastrointestinal stress conditions.** Survivability of 12 *B. longum* strains under simulated gastrointestinal stress conditions was tested *in vitro* and calculated based on the number of viable cells before and after incubation in simulated solutions (A–D), or under stressed and non-stressed conditions (E–F). (A) Simulated gastric fluid was prepared using 0.5% of pepsin(w/v), 0.5% of NaCl(w/v) and 6N HCl to adjust pH to 3 and the bacteria strains were incubated for 2 h. (B and C) Acidic conditions were prepared using MRS medium supplemented with 0.05% L-cysteine, adjusted to pH 3 with 6N HCl, and bacteria strains were incubated for 1 or 2 h. (D) Bile salt stress was evaluated using MRSc medium supplemented with 0.2% bile salts, and bacterial strains were incubated for 4 h. (E) Lysozyme tolerance was tested in electrolyte solution with or without lysozyme, and bacterial strains were incubated for 2 h. (F) Osmotic stress conditions were generated by supplementing MRSc medium with NaCl, and bacterial strains were incubated for 48 h. Values represent the mean ± standard error of the mean (SEM) of three independent replicates. Different letters indicate significant differences among strains (P < 0.05). Asterisks indicate significance: *P < 0.05, **P < 0.01, ***P < 0.001, and ****P < 0.0001.

Acid tolerance was further evaluated by incubating the isolates in growth medium adjusted to pH 3 for 1 h or 2 h, simulating postprandial gastric conditions, reflecting the transient elevation of gastric pH following food intake (Fig. 3B and C). Under simulated postprandial gastric conditions (pH 3.0), all *B. longum* strains exhibited high survivability (>10% viability), indicating substantial resistance to physiologically relevant gastric acidity. Some strains, such as KCJ2K3504, KCJ2K3532, KCJ2K3574, KCJ2K3577, KCJ2K3591, KCJ2K3632, KCJ2K3648, and KCJ2K3671 exhibited relatively greater acid tolerance than the other isolates, with greater than 50% viability after 2 h incubation. Overall, *B. longum* isolates exhibited strong resistance to acidic environments, suggesting robust survivability during gastric digestion.

Bile salts are secreted by the liver and stored in the gallbladder and play an essential role in the emulsification of dietary lipids and exert antimicrobial activity in the gut environment (Tancharoenrat et al., 2022). Therefore, bile salt tolerance is considered a key functional trait of probiotics. Bile salt tolerance was assessed to evaluate the ability of the *B. longum* isolates to withstand antimicrobial bile components encountered in the small intestine. Bacterial growth was measured in MRSc medium supplemented with 0.2% of bile salts (Fig. 3D). Strains KCJ2K3504, KCJ2K3532, and KCJ2K3574 showed significantly higher viability under bile salt exposure, whereas strains KCJ2K3568, KCJ2K3577, KCJ2K3591, KCJ2K3671, and KCJ2K3673 exhibited lower viability compared to without bile salts. Nevertheless, all strains maintained viable counts exceeding 10^7^ CFU/mL under bile salt stress.

Lysozyme is an antimicrobial enzyme secreted primarily by the salivary glands that plays an important role in innate immunity by targeting a wide range of pathogens, including bacteria, viruses, and fungi (Khorshidian et al., 2022). In particular, it exerts an antimicrobial effect by hydrolyzing the β-1,3-glycosidic bond between N-acetylmuramic acid and N-acetylglucosamine in the peptidoglycan layer of Gram-positive bacteria (Khorshidian et al., 2022). Therefore, lysozyme resistance is an essential characteristic of probiotic strains to reflect their ability to survive in the upper gastrointestinal tract. All *B. longum* strains exhibited substantial resistance to lysozyme exposure, with strain KCJ2K3504 showing significantly higher viability compared to other strains and control conditions without lysozyme (Fig. 3E). Nonetheless, all strains showed strong resistance to lysozyme exposure.

Probiotics can be exposed to osmotic stress during industrial processing, storage, and the digestion process in the intestinal lumen, which can disrupt bacterial homeostasis, caused by excessive water influx, bacterial lysis, and dehydration (Bustos et al., 2025). Osmotic tolerance of *B. longum* strains was evaluated by incubating the isolates in MRSc media supplemented with increasing salt concentrations (0, 1, 2, and 3%) to simulate osmotic stress. Most strains showed normal growth at 0% and 1% NaCl, whereas growth inhibition was observed at 2 and 3% NaCl across all isolates (Fig. 3F).

Collectively, all 12 *B. longum s*trains exhibited tolerance to SGF, acidic pH, bile salts, lysozyme, and osmotic stress conditions. Notably, *B. longum* KCJ2K3504, KCJ2K3532, and KCJ2K3574 strains consistently showed superior survivability across all stress tolerance assays. These results indicate that the potential *B. longum* strains derived from healthy calves possess robust GIT resilience, supporting their potential as viable probiotic candidates capable of surviving oral administration and GIT passage.

### 3.4 Colonization and adhesion properties of *B. longum* strains under mucus-associated conditions

For probiotic bacteria to exert beneficial effects in the GIT, they must not only survive from digestive stresses but also adhere to and colonize intestinal mucosal surfaces (Han et al., 2021). Adhesion to the host epithelium represents a critical initial step in colonization, enabling host-microbe interactions and persistence within the GIT environment (Nataraj et al., 2025). Colonization related traits are influenced by multiple bacterial surface properties, such as cell surface hydrophobicity, which facilitates initial contact with mucosal surfaces, and auto aggregation, which promotes microcolony formation to protect them from GIT stressors (Manvelyan et al., 2023; Lee et al., 2024). In addition, the ability to bind to intestinal mucosal surfaces as well as association with dietary fibers and undigested feed particles by forming biofilms contributes to bacterial persistence under the dynamic conditions of the GIT (Flemming et al., 2023; Liu et al., 2023). Accordingly, four *in vitro* assays were performed to evaluate colonization and adhesion related properties of *B. longum* strains under simulated mucus-related conditions.

Cell surface hydrophobicity was first evaluated as an indicator of bacterial affinity for non-polar surfaces and its potential contribution to adhesion and biofilm formation (Behbahani et al., 2024). *B. longum* strains were incubated with xylene to assess hydrophobicity. Among the isolates, KCJ2K3648 and KCJ2K3673 exhibited the greatest hydrophobicity values (95.5 and 96.3%, respectively), and KCJ2K3504, KCJ2K3522, KCJ2K3564, KCJ2K3568, KCJ2K3574, KCJ2K3648, KCJ2K3671, and KCJ2K3673 exhibited hydrophobicity levels exceeding 50% (Fig. 4A). These results indicate substantial strain-dependent variability in surface properties among the *B. longum* that may affect on colonization efficiency in the GIT.

**Fig. 4:**
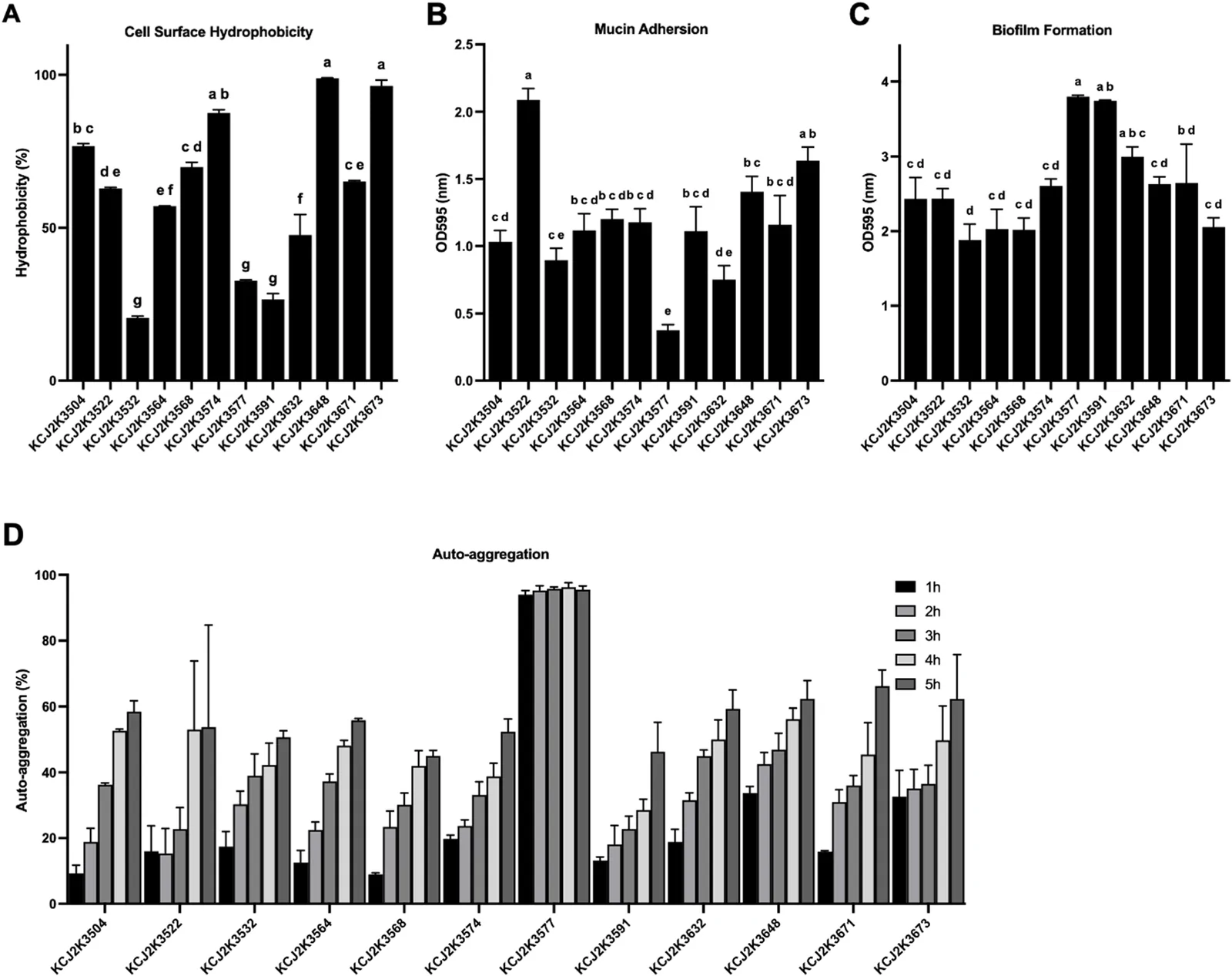
**Adhesion- and colonization-related properties of *B. longum* strains isolated from healthy calves.** The adhesion and colonization properties of 12 *B. longum* strains were tested *in vitro*. (A) Cell surface hydrophobicity was evaluated by measuring bacterial adhesion to xylene after 1 h incubation. Mucin adhesion capacity (B) and biofilm formation ability (C) were evaluated by measuring the adhesion to mucin-coated well for 2 and 48 h of incubation, respectively. (D) Auto-aggregation ability was determined by assessing the ability of bacterial cells to aggregate, and measurements were taken after 5 h. Values represent means ± standard error of the mean (SEM). Different letters indicate statistical differences among strains (*P* < 0.05).

Because the intestinal epithelium is covered by a mucus layer composed primarily of mucin glycoproteins, which serve as the first physical barrier and interface between the host and gut microbes (Breugelmans et al., 2022), the mucin-binding ability of *B. longum* strains was evaluated using mucin-coated microplates followed by crystal violet staining. After 2 h of incubation, KCJ2K3522 showed the highest mucin adherence, whereas KCJ2K3577 showed the lowest mucus-binding capacity compared to other isolates (Fig. 4B). With the exception of KCJ2K3577, all strains demonstrated measurable adhesion to mucus-coated surfaces, indicating their capacity to interact with intestinal mucus.

In the GIT, biofilm formation can promote bacterial survival by providing defense against host enzymes, antimicrobial molecules, and mechanistic flushing, while also contributing to the competitive exclusion of pathogenic bacteria (Liu et al., 2021). Biofilm formation was further assessed to evaluate the ability of *B. longum* strains to establish stable microbial communities under mucus-associated conditions. Biofilms were quantified using crystal violet staining in mucin-coated microplates. All strains formed biofilms, with KCJ2K3577 exhibiting significantly greater biofilm-forming capacity than the other isolates (Fig. 4C). These results suggest that the evaluated *B. longum* strains possess the ability to form biofilms in the gut mucosal environment.

Auto-aggregation, which reflects the ability of bacterial cells to adhere to each other and contributes to colonization and biofilm development on mucosal surfaces, was evaluated over a 5 h incubation period (Fig. 4D) enables bacteria to form stable microbial communities by protecting them from environmental stressors. KCJ2K3577 showed most rapid and pronounced auto-aggregation, reaching an aggregation rate of 94.0%. In contrast, the remaining strains displayed a gradual increase in auto-aggregation over time, with aggregation rates ranging from 45.0% to 66.2% after incubation. These findings demonstrate variability in auto-aggregation capacity among the *B. longum* strains.

Collectively, the 12 *B. longum* strains isolated from healthy calves exhibited favorable colonization-related properties. Most strains demonstrated strong cell surface hydrophobicity and auto-aggregation capacity, while mucin adhesion assay confirmed their ability to interact with the intestinal mucus layer. Furthermore, all strains displayed biofilm-forming ability under mucus-associated conditions. These findings suggest the potential of *B. longum* to colonize the GIT following oral administration and contribute to probiotic functionality.

### 3.5 Inhibitory effects of *B. longum* strains against *Salmonella* spp

Probiotics can contribute to host health not only by modulating the gut microbiota but also inhibiting the growth and colonization of enteric pathogens (Zhai et al., 2024; Fan et al., 2021; Cizeikiene and Jagelaviciute, 2021). To evaluate the potential antimicrobial activity of *B. longum*, we targeted *Salmonella* because they represent one of the most prevalent and economically significant zoonotic bacteria in cattle production systems, with direct implications for both animal health and preharvest food safety. In particular, *S.* Typhimurium and Dublin differ in host range and disease manifestation, allowing evaluation of probiotic efficacy against both broad-host enteric infection and cattle adapted invasive salmonellosis (Bentum et al., 2025). Accordingly, we evaluated the inhibitory potential of *B. longum* strains against two pathogenic *Salmonella* strains, *S.* Typhimurium KCJ187 and *S.* Dublin KCJ2K3839.

Lactic acid bacteria, including *Bifidobacterium* and *Lactobacillus,* are known to produce antimicrobial compounds such as organic acids and bacteriocin-like peptides that suppress pathogen growth (Goa et al., 2022; Zhang et al., 2025b). To assess whether *B. longum* produces antimicrobial metabolites, we collected cell-free supernatants from *B. longum* culture (pH = 4.5 ± 0.05) and used for agar well diffusion assay. Clear inhibition zones were observed surrounding wells containing *B. longum* supernatants, indicating antimicrobial activity against both *Salmonella* strains (Fig. 5A), but it was significantly reduced when the cell-free supernatant was neutralized (Supplementary Fig. 1). All *B. longum* isolates showed strong inhibitory effects against *S.* Typhimurium (Fig. 5B) and *S.* Dublin (Fig. 5C).

**Fig. 5:**
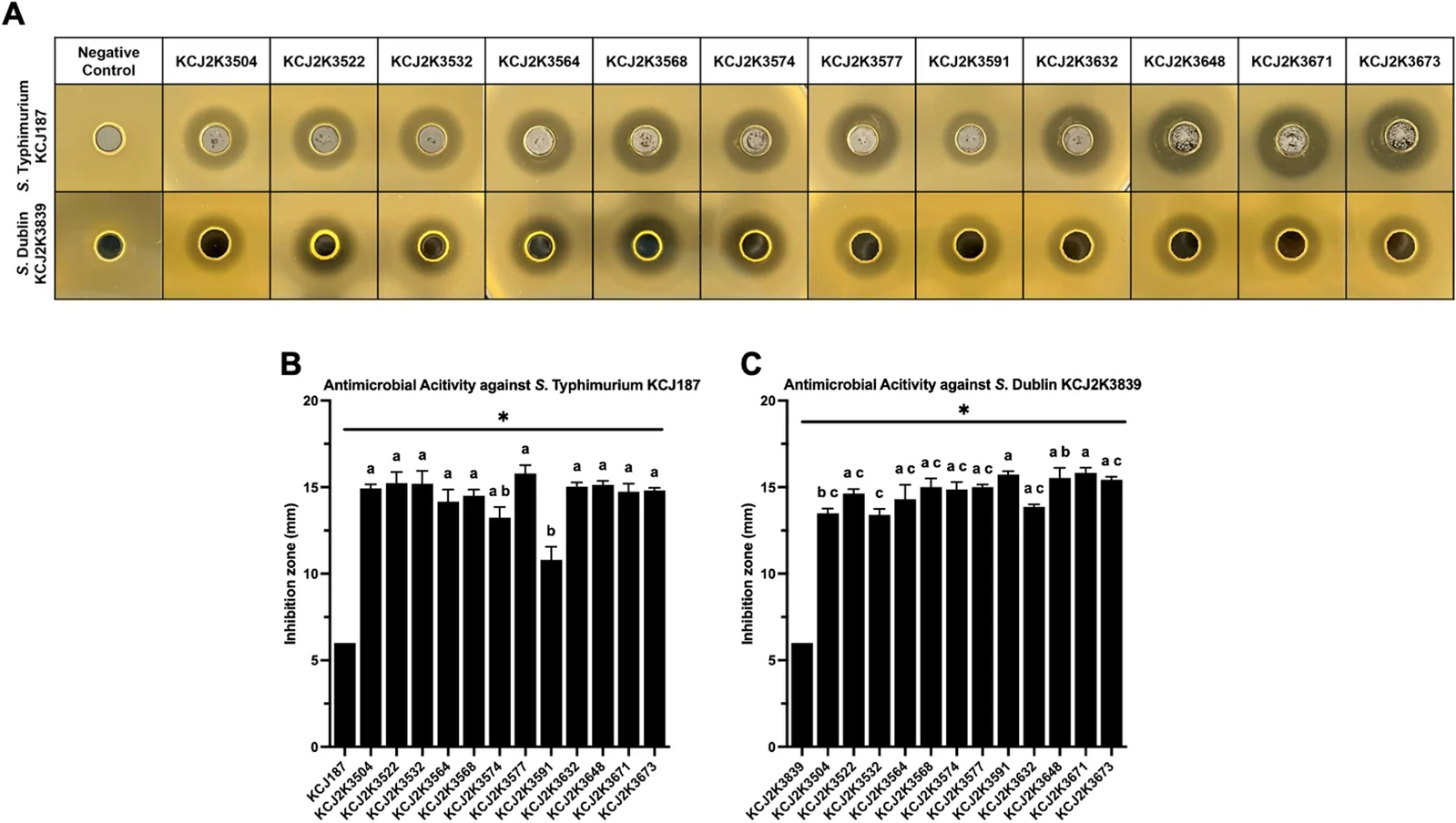
**Antimicrobial activity of *B. longum* strains against *Salmonella* Typhimurium and Dublin.** Antimicrobial activity of *B. longum* strains was determined by applying cell-free supernatants from bacterial cultures onto sTSA, whereas supernatants from *Salmonella* cultures were used as the negative control. (A) Representative pictures of inhibition zone. (B, C) The diameter of inhibition zone indicates antimicrobial activity against *S.* Typhimurium KCJ187 and *S.* Dublin KCJ2K3839. Values represent means ± standard error of the mean (SEM). Different letters indicate significant differences among the strains (*P* < 0.05). The asterisks (*) indicate significant differences between the control and each *B. longum* strain, determined by one-way ANOVA followed by Dunnett's multiple comparison test (*P* < 0.05).

In addition, we conducted competition assay to test if *B. longum* may suppress *S.* Typhimurium and *S.* Dublin strains when they were co-cultured. In monoculture, both *S.* Typhimurium and *S.* Dublin reached approximately 10^8^ CFU/mL after 48 h incubation in MRSc media. In contrast, co-culture with *B. longum* strains significantly suppressed the growth of both *Salmonella* serovars. Specifically, *B. longum* KCJ2K3532, KCJ2K3568, and KCJ2K3577 completely inhibited the growth of *S.* Typhimurium (Fig. 6A), while *B. longum* KCJ2K3504, KCJ2K3522, KCJ2K3564, KCJ2K3574, KCJ2K3591, KCJ2K3632, and KCJ2K3673 completely inhibited the growth of *S.* Dublin (Fig. 6B), indicating *B. longum* possess antimicrobial activity against *Salmonella.* Taken together, *B. longum* strains exhibited a pronounced inhibitory activity against *S.* Typhimurium and Dublin, probably associated with organic acid production*.* These results highlight the potential of calf-derived *B. longum* strains as probiotic candidates for controlling S*almonella* in livestock production systems.

**Fig. 6:**
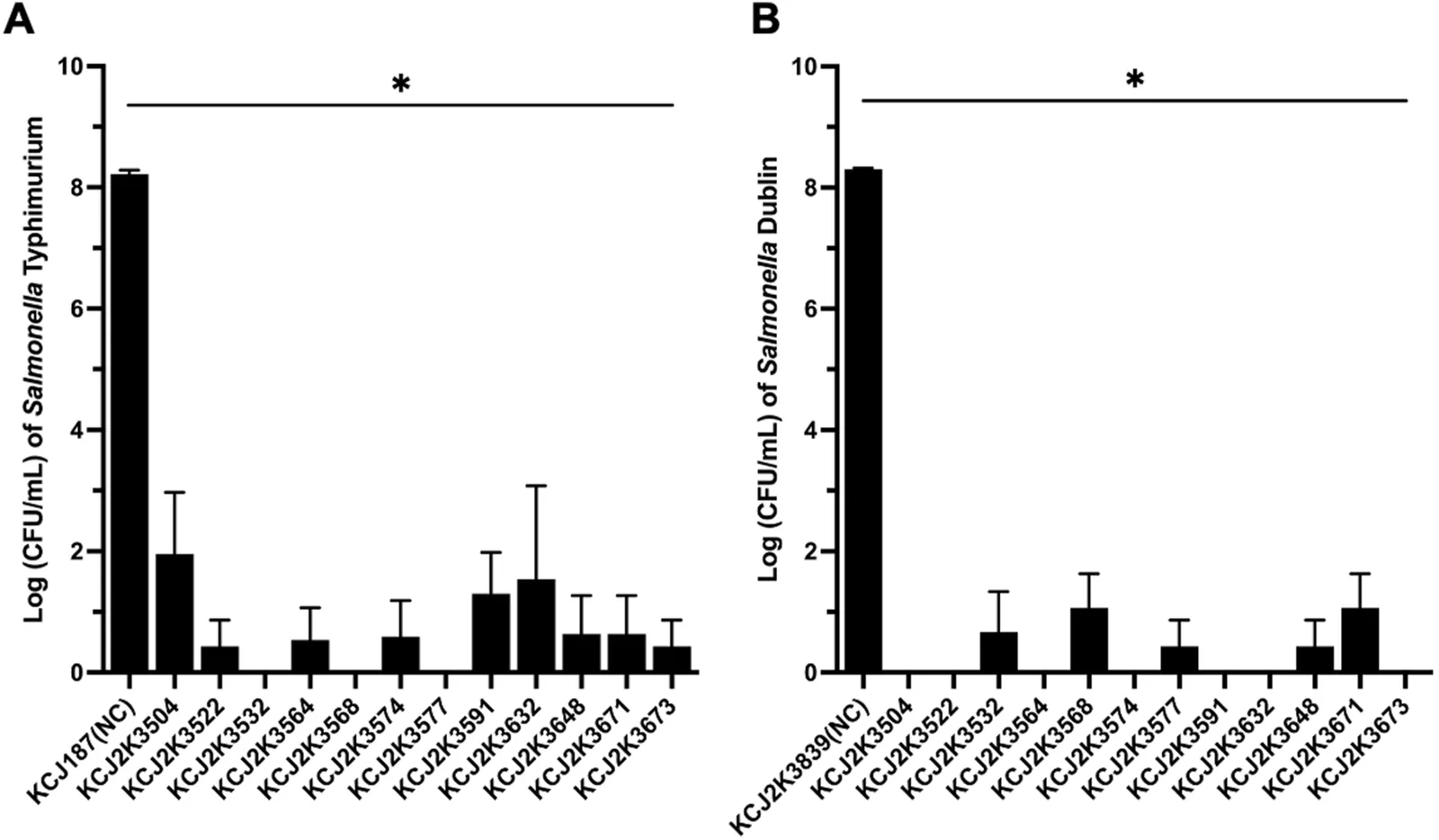
**Bacterial competition assay of *B. longum* strains against *Salmonella* Typhimurium and Dublin.** The number of surviving *Salmonella enterica* spp. after co-culture with each *B. longum* strain for 48 h in MRSc medium. (A, B) The inhibitory effect of *B. longum* strains against *S.* Typhimurium KCJ187 and *S.* Dublin KCJ2K3839 was evaluated. Negative control (NC) cultures were prepared without co-incubation with *B. longum* strains. Values represent means ± standard error of the mean (SEM). The asterisks (*) indicate significant differences between NC and each *B. longum* strain, determined by one-way ANOVA followed by Dunnett's multiple comparison test (*P* < 0.05).

### 3.6 Antibiotic resistance of probiotic candidates

The presence of antimicrobial genes (ARGs) in probiotic candidates represents a critical safety issue due to the potential for horizontal gene transfer within the GIT. A comprehensive *in silico* analysis was conducted to evaluate the genomic safety of the *B. longum* isolates with a focus on antimicrobial resistance. ARGs were detected in 10 of the 12 *B. longum* strains (Fig. 7) but two isolates, KCJ2K3532 and KCJ2K3574, did not carry any detectable ARGs. Among the remaining isolates, KCJ2K3568 encoded the highest number of ARGs (n = 9), followed by KCJ2K3591 (n = 8). The majority of detected ARGs across the isolates corresponded to intrinsic or genus-associated resistance determinants commonly reported in *Bifidobacterium* spp., such as *rpoB* and *ileS*, which are commonly reported and not associated with mobile genetic elements. Additional resistance genes identified across the isolates included *dfrF*, which encodes a dihydrofolate reductase associated with diaminopyrimidine resistance, as well as *tetW*, a ribosomal protection protein conferring tetracycline resistance. Although *tetU* was detected in one isolate, this gene has been shown not to function as a tetracycline resistance determinant but instead corresponds to the misannotated 3′ region of a rolling-circle replication initiator protein. Other ARGs included *sat-4*, associated with streptothricin acetyltransferase (Jacob et al., 1994), *APH(3′)-IIIa* and *aad(6)*, both encoding aminoglycoside-modifying enzymes, and *erm(49)*, an rRNA methyltransferase conferring macrolide-lincosamide-streptogramin resistance, previously identified in probiotic *Bifidobacterium* strains; however, these genes have been reported to be predominantly chromosomal and to pose a low risk for horizontal gene transfer. Importantly, no clear associations were observed between ARG content (Fig. 7) and phylogenetic clustering (Fig. 1). These data indicate that the calf-derived *B. longum* strains exhibit genomic safety profiles, particularly KCJ2K3532 and KCJ2K3574, and support their suitability as probiotic candidates.

**Fig. 7:**
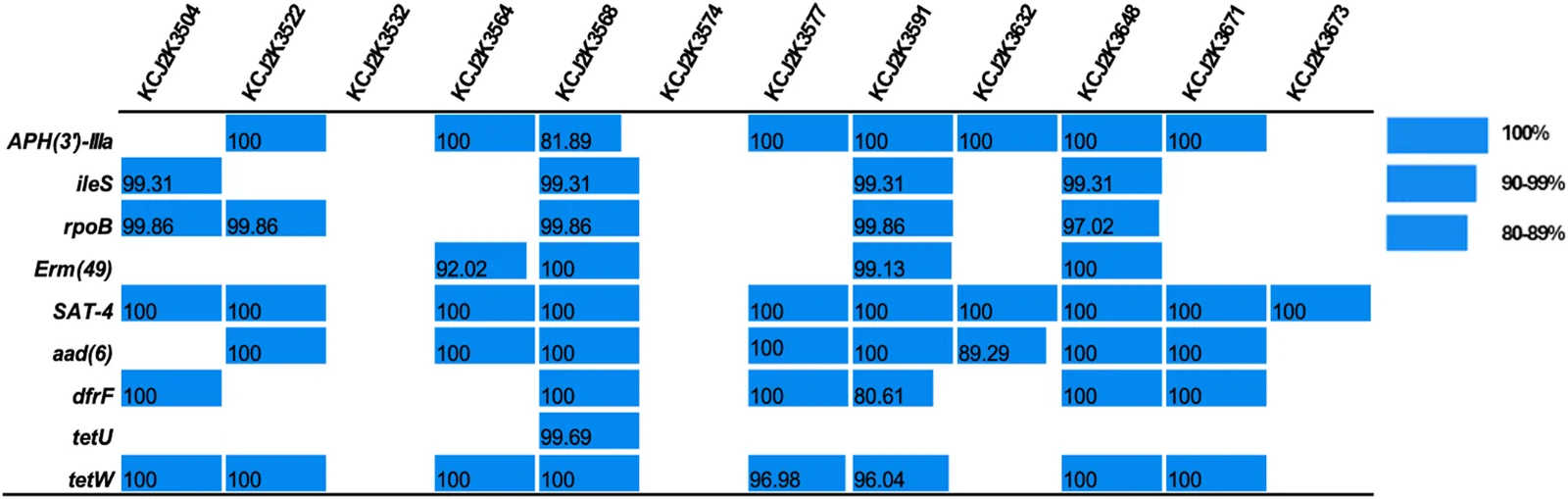
**Antimicrobial resistance gene profiles of *B. longum* isolates.** Antimicrobial resistance genes (ARGs) were identified using the CARD database. For each detected ARG, the length and number of blue bars within each cell represent the sequence identity (%) to the corresponding reference gene, indicating the degree of sequence similarity.

## 4 Discussion

This study presents an integrated genomic and functional evaluation of *B. longum* strains isolated from healthy pre-weaned dairy calves and demonstrates their potential as host-associated probiotic candidates, inferred from phylogenetic divergence and *in vitro* functional characterization. Comparative phylogenomic analyses revealed that the isolates are genetically distinct from publicly available human- or other livestock-derived *B. longum* strains, highlighting lineage-level divergence associated with host origin. The isolates exhibited a range of probiotic-relevant phenotypes, including resilience to gastrointestinal stressors, diverse colonization-related traits, and antimicrobial activity against *Salmonella* serovars.

Antimicrobial activity is critical functional attribute of probiotics intended for pathogen control in livestock, particularly in neonatal calves, where enteric infections such as salmonellosis pose substantial health and economic burdens. As shown in Fig. 1 and Table 1, comparative genomic analyses revealed multiple features consistent with probiotic functionality, including metabolic pathways associated with carbohydrate utilization and stress response; however, no clearly annotated bacteriocin biosynthetic gene clusters were identified in the *B. longum* genomes. Previous studies have reported bacteriocin-like antimicrobial peptides in *B. longum*, including longumicin-related compounds (Du et al., 2025), as well as cryptic ribosomally synthesized peptides identified through functional assays rather than genomic prediction (Liu et al., 2015). Despite the absence of clearly annotated bacteriocin-associated genes, the *B. longum* isolates exhibited pronounced inhibitory activity against *Salmonella* serovars (Fig. 5, Fig. 6). Importantly, antimicrobial activity of the cell-free supernatants was significantly reduced following neutralization (Supplementary Fig. 1), indicating that the observed inhibition was primarily pH-dependent and likely associated with organic acid production. This finding is consistent with previous reports demonstrating organic acid mediated inhibition of enteric pathogens by *Bifidobacteria* (El-Mokhtar et al., 2020; Marianelli et al., 2010). Similarly, a fermented sour wort marinade containing *Pediococcus acidilactici* PA-2 reduced *S*. Typhimurium and *Listeria monocytogenes* in refrigerated raw chicken through organic acid-mediated acidification (pH 3.8-4.5), supporting acidification as an effective antimicrobial mechanism of lactic acid bacteria (Mukherjee et al., 2026). In addition to the antimicrobial activity observed with cell-free supernatants (Fig. 5), co-culture competition assays demonstrated strong competitive exclusion of *Salmonella* (Fig. 6). Similarly, *Pediococcus acidilactici* IAH-5 reduced *S*. Typhimurium adhesion to Caco-2 cells, supporting co-culture inhibition against pathogens (Vasiee et al., 2020). Collectively, these findings suggest that the antagonistic activity of the calf-derived *B. longum* isolates against *Salmonella* is probably associated with organic acid production and environmental acidification, while other mechanisms, including competitive interactions and exopolysaccharide-associated effects (Jeong et al., 2017), may also contribute.

A prerequisite for probiotic performance is the ability to remain viable during upper GIT passage, where exposure to gastric enzymes and acidity, followed by bile-mediated stress and host antimicrobial factors, represents a major challenge for bacterial persistence (Gan et al., 2026). *In vitro* assays demonstrated that calf-derived *B. longum* strains possess key functional traits required for effective probiotic performance, including resistance to gastric acidity, bile salts, lysozyme, and osmotic stress, with all strains remaining above 10^7^ CFU/ml under these challenges (Fig. 3). The survivability observed here is consistent with prior work showing the *B. longum* were tolerant to acid challenges and viable during low pH exposure (Sánchez et al., 2007). These resilience traits are particularly relevant for neonatal calves, whose gastrointestinal environment undergoes rapid physiological and dietary changes during early development. Likewise, the ability of our isolates to remain viable under bile salt challenge may align with the bile adaptation mechanisms such as bile-protection systems and bile salt hydrolase, encoded by the *bsh* gene (Fig. 1 and Table 1), associated traits described in *Bifidobacterium* (Ruiz et al., 2012). Notably, we observed strain-to-strain variation across *in vitro* survival assays, with a subset of isolates, including KCJ2K3504, KCJ2K3532, and KCJ2K3574, demonstrated consistently superior tolerance across SGF, acid, bile, NaCl, suggesting enhanced adaptive capacity for survival during GIT passage. These data may be consistent with strain-level genomic heterogeneity shown in Fig. 1A, indicating that survivability is strain dependent and providing a reason for prioritizing these isolates for downstream *in vivo* validation and functional efficacy studies in calves. Building on these findings, integration of the antimicrobial activity (Fig. 5, Fig. 6), genomic safety profiles (Fig. 7), stress tolerance (Fig. 3), and colonization capacity (Fig. 4) suggests that KCJ2K3532 and KCJ2K3574 may be promising candidates for further evaluation. Both strains lacked detectable ARGs and showed strong antimicrobial activity against *Salmonella*. Although further studies such as comprehensive safety assessments and evaluation of host responses, are required, these findings provide a basis for prioritizing these strains for future in *vivo* studies.

Beyond survival during GIT passage, effective probiotics must establish persistence within the gut through colonization strategies. As shown in Fig. 4, we observed the *B. longum* isolates exhibited diverse colonization-related phenotypes, including cell surface hydrophobicity, auto-aggregation, mucin adhesion, and biofilm formation that enable stable association with host-associated niches, including the mucosal layer, epithelial surfaces, and dietary substrates present in the intestinal lumen. High surface hydrophobicity observed in several isolates is consistent with previous reports linking hydrophobic cell surfaces to enhanced initial adhesion and biofilm development in *Bifidobacterium* and other probiotic taxa (Xu et al., 2009). Likewise, strain-dependent variability in mucin-binding capacity mirrors earlier observations that adhesion to mucus is highly strain specific and influenced by surface-associated proteins, exopolysaccharides, and glycan-recognition mechanisms (Westermann et al., 2016; Lu et al., 2022). Notably, some strains, including KCJ2K3577, displayed strong biofilm formation and aggregation despite moderate mucin adherence, suggesting alternative colonization strategies that may involve attachment to dietary fibers or undigested feed particles (Kelly et al., 2020). The pronounced auto-aggregation observed in KCJ2K3577 further support the formation of stable microbial microcolonies, which can enhance resistance to mechanical flushing and environmental stress while promoting competitive exclusion of pathogens within the gut lumen (Chiang et al., 2024).

*B. longum* appears to play a central role in shaping gut function and host development in dairy calves. *Bifidobacterium* produces lactic and acetic acids that acidify the large intestine, thereby suppressing putrefactive and pathogenic bacteria and promoting intestinal health (Servin, 2004). Recent calf studies further demonstrate that a *Bifidobacterium*-dominated hindgut microbiota is associated with improved growth phenotypes, with fecal microbiota transplantation experiments confirming that *Bifidobacterium* contributes to enhanced host growth and hindgut development in early life. Follow-up work showed that core hindgut *Bifidobacterium* regulates microbial functions and host metabolism by stimulating carbohydrate degradation, glycolysis, and protein and propionate biosynthesis while suppressing methane production; isolated *B. longum* strains from calves also promoted host growth, immunity, antioxidative capacity, and intestinal development in murine models (Zhuang et al., 2025). Associations between *B. longum* and healthy lactating cows have likewise been observed, with fecal transplantation experiments supporting its functional contribution to host health (Hsieh et al., 2023). In addition, *B. longum* subsp. *infantis* showed benefits in alleviating constipation through gut microbiota modulation in mice (Fu et al., 2024). Nutritional interventions could further support the importance of *Bifidobacterium*. Study found that fructo-oligosaccharide supplementation in nursing calves enhanced growth while promoting persistence of *Bifidobacterium* and maturation of the hindgut microbiome, with the majority identified as *B. longum* (Gao et al., 2024). Together, these findings suggest that *B. longum* functions as an important early-life microbial component in dairy calves, supporting gut ecosystem stability, metabolic maturation, and host growth and health, making it a potential probiotic candidate.

In conclusion, this study demonstrates that host-associated *B. longum* strains isolated from healthy dairy calves exhibit probiotic traits, including gastrointestinal resilience, colonization potential, antimicrobial activity against *Salmonella* spp., and favorable genomic safety profiles. As the probiotic safety assessment was based on *in silico* genomic screening, further phenotypic validation, including comprehensive safety and host-response assessments, is required. Future studies should focus on *in vivo* validation of colonization and pathogen inhibition in calves, purification and characterization of antimicrobial compounds, and integration of transcriptomic or metabolomic approaches to elucidate active mechanisms. Collectively, these efforts will advance the development of calf-specific probiotics as targeted biotherapeutics to enhance gut health and reduce pathogen burden in livestock production systems.

## Ethics declaration

This study was conducted in accordance with the ARRIVE (Animal Research: Reporting of In Vivo Experiments) guidelines. This study was approved by the University of Florida.

## Data availability

Raw reads are available on the Sequence Read Archive of the National Center of Biotechnology Information (NCBI) under the BioProject number PRJNA1420936.

## Contributions

KCJ and MK acquired funding. SK, MK, MK, and KCJ conceptualized. YZ and BC collected samples. YZ, BC, JC, KK and MK analyzed, collected, and visualized data. KCJ provided resources. MK and KCJ supervised and validated. BC, YZ, JC, MK, MK, YK, SO, SK and KCJ wrote the first draft. All authors wrote and reviewed the main manuscript.

## Declaration of competing interest

The authors declare that they have no known competing financial interests or personal relationships that could have appeared to influence the work reported in this paper.
