# Supplementary material for: Host-associated Bifidobacterium longum exhibit gastrointestinal resilience and antagonistic activity against Salmonella serovars
Source: Curr Res Food Sci. 2026 Sep 22;13:101580. doi: 10.1016/j.crfs.2026.101580 (PMC13634303; doi:10.1016/j.crfs.2026.101580)
Supplement: Supplementary file 1 [file mmc1.docx]

**Supplementary information**

**Host-associated *Bifidobacterium longum* exhibit gastrointestinal resilience and antagonistic activity against *Salmonella* serovars**

Bugil Choi^1,2,3*^, Yuting Zhai^1,2*^, Suyeun Noh^1,2^, Jonathan Crabill^1^, Katherine Kim^1,2^, Miju Kim^4^, Sangnam Oh^5^, Younghoon Kim^6^, Sam-Churl Kim^3^, Min-Jin Kwak^1,2,7#^ and Kwangcheol C. Jeong^1,2#^

^1^Emerging Pathogens Institute, University of Florida, Gainesville, FL 32611, United States

^2^Department of Animal Sciences, IFAS, University of Florida, Gainesville, FL 32611, United States

^3^Division of Applied Life Science (BK21Four, Institute of Agriculture & Life Science), Gyeongsang National University, Jinju 52828, Republic of Korea

^4^Department of Food Science & Biotechnology, Kyung Hee University, Yongin 17104, Republic of Korea

^5^Department of Functional Food and Biotechnology, Jeonju University, Jeonju 55069, Republic of Korea

^6^Department of Agricultural Biotechnology and Research Institute of Agriculture and Life Science, Seoul National University, Seoul 08826, Republic of Korea

^7^Department of Forest Products and Biotechnology, Kookmin University, Seoul 02707, Republic of Korea

*These authors contributed equally.

^#^Corresponding authors:

Dr. Min-Jin Kwak, Department of Forest Products and Biotechnology, Kookmin University, Seoul 02707, Republic of Korea, ics1051@kookmin.ac.kr

Dr. Kwangcheol C. Jeong, Department of Animal Sciences, Emerging Pathogens Institute, University of Florida, Gainesville, FL, United States, [kcjeong@ufl.edu](mailto:kcjeong@ufl.edu)

**Supplementary Table 1. Strain and sequencing information**

| **Strain** | **Source** | **Calf tag** | **Age (Days)** | **Mean depth (×)** | **Assembly covered (%)** | **N50 (bp)** | **GC (%)** | **Completeness (%)** | **Contamination (%)** | **SRA accession** |
| --- | --- | --- | --- | --- | --- | --- | --- | --- | --- | --- |
| KCJ2K3504 | Healthy Holstein calf | 10815 | 14 | 34.28 | 99.98 | 26,941 | 59.67 | 96.64 | 1.86 | SRR37195610 |
| KCJ2K3522 | Healthy Holstein calf | 10826 | 9 | 15.43 | 99.95 | 3,722 | 59.64 | 89.36 | 3.44 | SRR37195609 |
| KCJ2K3532 | Healthy Holstein calf | 10833 | 3 | 9.63 | 99.99 | 908 | 60.52 | 54.75 | 0.62 | SRR37195606 |
| KCJ2K3564 | Healthy Holstein calf | 10839 | 14 | 26.81 | 99.59 | 2,952 | 59.33 | 82.07 | 4.17 | SRR37195605 |
| KCJ2K3568 | Healthy Holstein calf | 10839 | 28 | 56.9 | 99.79 | 10,641 | 59.55 | 89.28 | 0.75 | SRR37195604 |
| KCJ2K3574 | Healthy Holstein calf | 10842 | 3 | 16.59 | 99.67 | 915 | 58.44 | 41.24 | 0.86 | SRR37195603 |
| KCJ2K3577 | Healthy Holstein calf | 10842 | 3 | 29.1 | 99.69 | 2,737 | 59.22 | 77.69 | 5.66 | SRR37195602 |
| KCJ2K3591 | Healthy Holstein calf | 10843 | 9 | 56.16 | 99.81 | 17,295 | 59.62 | 99.91 | 2.66 | SRR37195601 |
| KCJ2K3632 | Healthy Holstein calf | 10848 | 28 | 21.39 | 99.61 | 1,279 | 59.1 | 57.62 | 1.97 | SRR37195600 |
| KCJ2K3648 | Healthy Holstein calf | 10852 | 28 | 51.11 | 99.82 | 10,500 | 59.73 | 94.04 | 1.75 | SRR37195599 |
| KCJ2K3671 | Healthy Holstein calf | 10854 | 9 | 22.25 | 99.64 | 1,566 | 59.2 | 70.45 | 2.19 | SRR37195608 |
| KCJ2K3673 | Healthy Holstein calf | 10854 | 9 | 15.66 | 99.78 | 820 | 58.9 | 32.58 | 1.43 | SRR37195607 |

**Supplementary Figure 1. Antimicrobial activity of *B. longum* strains against *Salmonella* Typhimurium and *Salmonella* Dublin at natural pH.** Agar well diffusion assay showing the inhibitory activity of *B. longum* culture supernatants at their natural pH against *S.* Typhimurium and *S.* Dublin. MRSc served as the negative control. Antimicrobial activity was evaluated based on the presence of inhibition zones surrounding the wells.
